# Endemic *Ehrlichia canis* infection in a dog from Ireland: case report

**DOI:** 10.1186/s13620-026-00357-z

**Published:** 2026-08-14

**Authors:** Julia McCarthy, Thomas Peter Large, Benoît Cuq

**Affiliations:** 1 School of Veterinary Medicine, University College Dublin, Belfield Co, Dublin, Ireland; 2 Primrose Hill Veterinary Clinic, Ophthalmology Referral Service, 1 Church Place, Sallynoggin, Co., Dublin, Ireland

**Keywords:** Canine monocytic ehrlichiosis, Ocular, Rhipicephalus sanguineus, Tick-borne disease, Case report

## Abstract

**Background:**

Canine monocytic ehrlichiosis, caused by *Ehrlichia canis*, is a tick-borne disease of dogs. We report the first confirmed case of endemic *Ehrlichia canis* infection in a dog from Ireland, with no travel history.

**Case presentation:**

A six-year-old female neutered lurcher, presented with chronic fever, lethargy, polyuria, polydipsia, weight loss, thrombocytopenia, non-regenerative anaemia, hyperglobulinaemia, proteinuria, and ocular abnormalities including chorioretinitis and partial retinal detachment. Diagnosis was confirmed by enzyme-linked immunosorbent assays, indirect fluorescent antibody, and polymerase chain reactions. Treatment consisting of doxycycline with systemic corticosteroids resulted in resolution of associated clinical and clinicopathological abnormalities.

**Conclusions:**

This case underscores the importance of comprehensive diagnostics, tick surveillance, and a One Health approach to identify, prevent, and manage vector-borne pathogens in Ireland. The presence of potentially endemic *Ehrlichia canis* in this dog raises concern for local transmission, potentially via *Rhipicephalus sanguineus* tick.

## Background

Canine monocytic ehrlichiosis (CME), caused by *Ehrlichia canis*, is a tick-borne disease of dogs and other canid hosts, that can range in severity from asymptomatic to fatal [1, 2]. *E.canis* (family *Anaplasmataceae*, order *Rickettsiae*) is a small (0.5–1.5 μm), obligate intracellular, gram-negative bacterium that preferentially infects peripheral blood monocytes, and macrophages. It can also disseminate to target organs, including the bone marrow, lymph nodes, spleen and liver [1, 2]. *Rhipicephalus sanguineus*, a tick, is the main vector for *E.canis* transmission [1–3]. Dogs are the primary reservoir hosts, although other *canids* such as foxes, wolves, and jackals may also serve as reservoirs [1, 4–6].

Diagnosis of *E. canis* infection relies on compatible clinical signs and supportive laboratory tests including enzyme-linked immunosorbent assay (ELISA), indirect fluorescent antibody (IFA), polymerase chain reaction (PCR) for DNA and direct identification of morulae on blood smear or cytology. Co-infections with multiple vector-borne pathogens are common in endemic regions and may exacerbate or mask clinical signs, complicating diagnosis. As many vector-borne diseases produce similar clinical signs and laboratory abnormalities, a thorough evaluation is required to identify or exclude concurrent infections [3].

Primarily a canine pathogen, *E.canis* can also cause clinical disease in cats [7]. Environmental changes and international pet movement have expanded the range of vector-borne pathogens [8]. This case report describes the first confirmed case of endemic *E.canis* infection in a dog from Ireland. It highlights the importance of surveillance for emerging tick-borne pathogens within a One Health framework and the impact of animal movement and climate change on the spread of infectious diseases to previously non-endemic regions.

## Case presentation

A six-year-old, 18 kg, female neutered lurcher from County Wicklow was referred to University College Dublin Veterinary Hospital– Small Animal Medicine Service for further investigation of a six-month history of waxing and waning pyrexia, gingival haemorrhage, polyuria, polydipsia, weight loss, lameness, generalized lymphadenomegaly, and lethargy. Bilateral abnormal ocular opacification with tapetal hyperreflectivity was also described. Sequential haematology and serum biochemistry profiles demonstrated persistent thrombocytopenia and non-regenerative anaemia over the course of the six months. The clinical signs and clinicopathological abnormalities had shown poor response to multiple empirical treatment courses, including amoxicillin-clavulanic acid and prednisolone administered at an immunomodulatory dose. The most recent course of amoxicillin-clavulanic acid (20 mg/kg orally every 12 h) and prednisolone (1 mg/kg orally every 24 h) were initiated two days prior to presentation. The dog had no relevant concurrent medical conditions and no history of travel outside Ireland. Preventative ectoparasite treatment was not up to date, and the dog was routinely exercised off lead in the Wicklow Mountains (County Wicklow, Ireland). The other dog in the household was clinically healthy, also had no travel history and was not up to date with preventative ectoparasite treatment.

On presentation, the dog was bright, alert, and responsive. Body condition score was 3/9, with mild to moderate generalized muscle loss. Cardiothoracic auscultation and abdominal palpation were unremarkable. Mucous membranes were pale pink with a capillary refill time of < 2 s, and peripheral pulses were strong and synchronous. No gingival haemorrhage was evident on the day of admission. Mild to moderate enlargement of the mandibular, prescapular and popliteal lymph nodes was present. Orthopaedic examination revealed no discomfort on joint manipulation and no detectable joint effusion. The dog was normothermic, and the remaining parameters of the general physical examination were within normal limits.

**Fig. 1:**
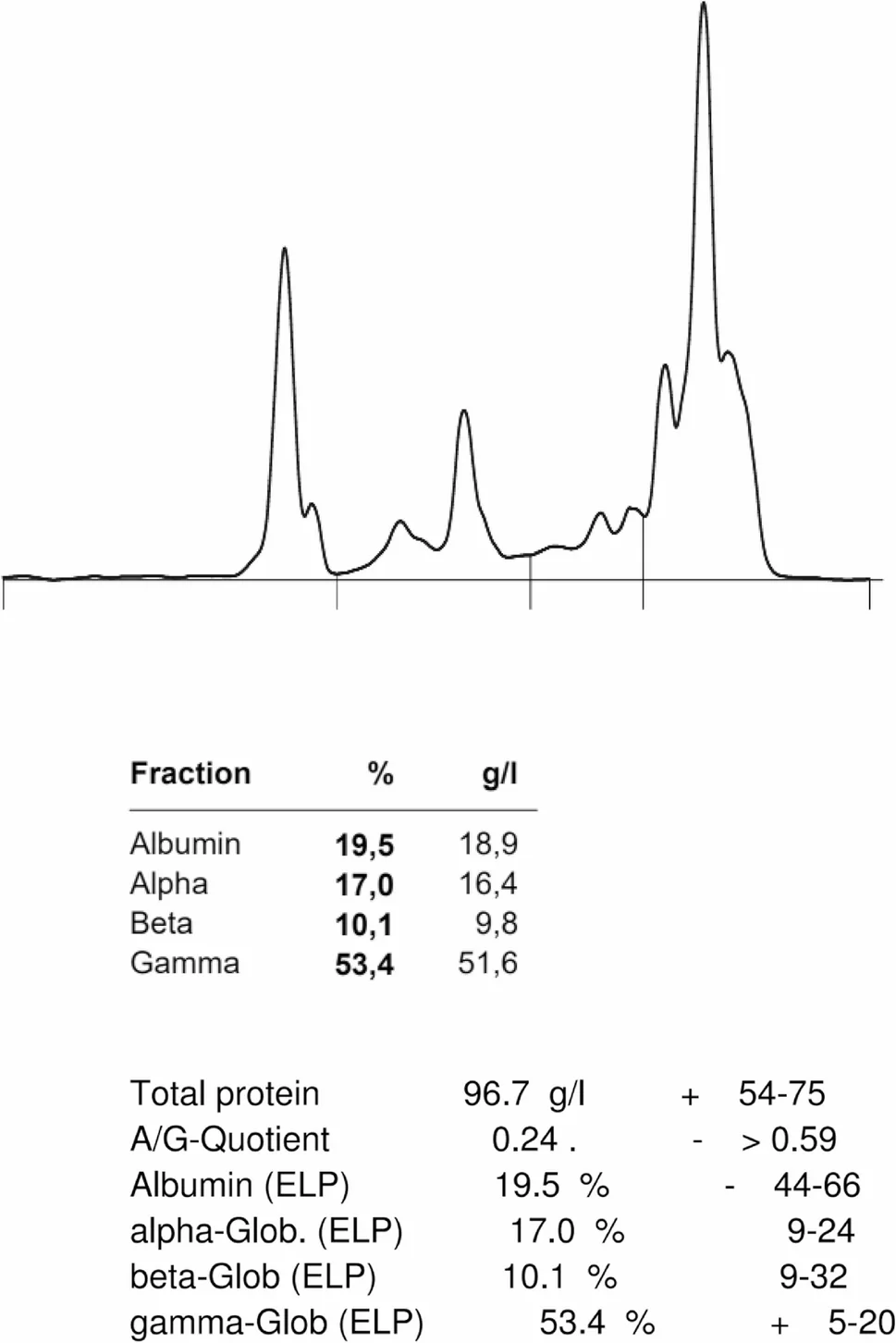
Serum protein electrophoresis demonstrating an oligoclonal gammopathy

Haematology revealed moderate non-regenerative anaemia and mild thrombocytopenia (Table 1). Peripheral blood smear showed no further abnormalities. Serum biochemistry demonstrated marked hyperglobulinaemia, and serum protein electrophoresis was therefore submitted to differentiate between a monoclonal and polyclonal gammopathy (Table 1). It revealed an oligoclonal gammopathy (Table 2; Fig. 1). Urinalysis revealed an isosthenuric urine specific gravity (1.012) with marked proteinuria (3+) (Table 2). The remainder of urine sediment analysis was within normal limits with no active sediment. The urine protein-to-creatinine ratio was markedly elevated at 4.7 (Reference interval: <0.5) (Table 2). Bacterial culture of the urine was negative. Urine protein electrophoresis was submitted for further characterisation of the proteinuria. Results did not support light-chain proteinuria but demonstrated predominant loss of albumin and other glomerular proteins, consistent with a glomerulopathy.

**Table 1:** Haematology and biochemistry in a six-year-old dog diagnosed with *E. canis*

	Pre Treatment	During treatment (10 days)	End of treatment (30 days)	One month post treatment	Six months post treatment	Ref. interval
Haematocrit	**0.20 L/L**	**0.24 L/L**	0.38 L/L	0.43 L/L	0.47 L/L	0.37–0.55 L/L
RBC	**2.91 × 10** ^**12**^ **/l**	**3.41 × 10** ^**12**^ **/l**	**5.31 × 10** ^**12**^ **/l**	6.11 × 10^12^/l	6.84 × 10^12^/l	*5.50–8.50 × 10* ^*12*^ */l*
Haemoglobin	**65 g/dl**	**81 g/dl**	165 g/dl	152 g/dl	163 g/dl	*120.00–180.00 g/dl*
MCHC	324 g/dl	340 g/dl	**440 g/dl**	354 g/dl	345 g/dl	*310.00–362.00 g/dl*
MCH	22.5 pg/l	23.9 pg/l	**31.1 pg/l**	24.8 pg/l	23.8 pg/l	*19.50–25.00 pg/l*
MCV	69.40 fl.	70.30 fl.	70.80 fl.	70.20 fl.	68.90 fl.	*60.00–77.00 fl.*
Reticulocytes	14.10 × 10^9^/l	**103.90 × 10** ^**9**^ **/l**	24.20 × 10^9^/l	43.30 × 10^9^/l	20.40 × 10^9^/l	*0–60 × 10* ^*9*^ */l*
Platelets	**117 × 10** ^**9**^ **/l**	**122 × 10** ^**9**^ **/l**	**787 × 10** ^**9**^ **/l**	235 × 10^9^/l	212 × 10^9^/l	*150–500 × 10* ^*9*^ */l*
MPV	**17.10 fl.**	9.50 fl.	**11.10 fl.**	8.30 fl.	9 fl.	*7.00–11.00 fl.*
WBC	**5.14 × 10** ^**9**^ **/l**	**5.86 × 10** ^**9**^ **/l**	11.80 × 10^9^/l	**3.07 × 10** ^**9**^ **/l**	**4.40 × 10** ^**9**^ **/l**	*6.00–17.00 × 10* ^*9*^ */l*
Neutrophil	4.32 × 10^9^/l	5.39 × 10^9^/l	11.05 × 10^9^/l	**2.58 × 10** ^**9**^ **/l**	3.34 × 10^9^/l	*3.00–11.50 × 10* ^*9*^ */l*
Bands	0	0	**0.12**	0	0	
Lymphocytes	**0.10 × 10** ^**9**^ **/l**	**0.12 × 10** ^**9**^ **/l**	**0.24 × 10** ^**9**^ **/l**	**0.22 × 10** ^**9**^ **/l**	**0.79 × 10** ^**9**^ **/l**	*1.00–3.60 × 10* ^*9*^ */l*
Monocytes	0.72 × 10^9^/l	0.35 × 10^9^/l	0.48 × 10^9^/l	0.25 × 10^9^/l	0.18 × 10^9^/l	*< 1.35 × 10* ^*9*^ */l*
Eosinophils	0 × 10^9^/l	0 × 10^9^/l	0 × 10^9^/l	0.03 × 10^9^/l	0.09 × 10^9^/l	*< 1.47 × 10* ^*9*^ */l*
Total protein	**96.50 g/l**	-	61.80 g/l	62.60 g/l	57 g/l	*54.00–71.00 g/l*
Albumin	25.90 g/l	**-**	34.20 g/l	32.70 g/l	g/l	*25.00–38.00 g/l*
Globulins	**70.60 g/l**	-	**27.60 g/l**	29.90 g/l	**26.40 g/l**	*28–42 g/l*
Urea	7.70 mmol/l	**-**	**13.10 mmol/l**	8.30 mmol/l	5.40 mmol/l	*3.60–8.60 mmol/l*
Creatinine	96.40 umol/l	-	62.79 umol/l	90.22 umol/l	70.59 umol/l	*20.00–120.00 umol/l*
Total calcium	2.40 mmol/l	-	2.50 mmol/l	2.60 mmol/l	2.60 mmol/l	*2.30–3.00 mmol/l*
Sodium	146 mmol/l	-	151 mmol/l	148 mmol/l	151 mmol/l	*137.00–151.00 mmol/l*
Inorganic phosphorus	1.80 mmol/l	-	1.80 mmol/l	1.80 mmol/l	1.20 mmol/l	*0.80–1.80 mmol/l*
Potassium	3.79 mmol/l	-	4.30 mmol/l	4.24 mmol/l	4.10 mmol/l	*3.70–5.80 mmol/l*
Glucose	5.88 mmol/l	-	3.66 mmol/l	**6.83 mmol/l**	5.87 mmol/l	*3.00–6.50 mmol/l*
Chloride	**119 mmol/l**	-	110 mmol/l	116 mmol/l	**118 mmol/l**	*105.00–117.00 mmol/l*
Total bilirubin	**0.50 umol/l**	**-**	**0 umol/l**	**0 umol/l**	**0.40 umol/l**	*0.90–10.00 umol/l*
ALP	**179.43 U/l**	-	**256.08 U/**l	40.36 U/l	43.19 U/l	*< 82.00 U/l*
ALT	**209 U/l**	**-**	**197 U/l**	**44 U/l**	**36 U/l**	*< 36.00 U/l*
GGT	5 U/l	-	16 U/l	8 U/l	0 U/l	*< 16.00 U/l*
AST	**81 U/l**	-	26 U/l	29 U/l	31 U/l	*< 37.00 U/l*
GLDH	**33.94 U/l**	-	**87.45 U/l**	4.02 U/l	2.79 U/l	*< 16.00 U/l*
Triglycerides	0.84 mmol/l	-	**8.02 mmol/l**	**1.81 mmol/l**	0.41 mmol/l	*0.11–1.69 mmol/l*
Cholesterol	5.18 mmol/l	-	**9.64 mmol/l**	**9.90 mmol/l**	**6.90 mmol/l**	*3.20–6.50 mmol/l*
Lipase	40 U/l	-	35 U/l	37 U/l	21 U/l	*< 130.00 U/l*

Bold text indicates abnormal values

*ALP* Alkaline phosphatase, *ALT* Alanine aminotransferase, *AST* Aspartate aminotransferase, *GGT* Gamma-glutamyl transferase, *GLDH* Glutamate dehydrogenase, *MCH* Mean corpuscular haemoglobin, *MCHC* Mean corpuscular haemoglobin concentration, *MCV* Mean corpuscular volume, *MPV* Mean platelet volume, *RBC* Red blood cell count, *Ref. interval* Reference interval

**Table 2:** Additional clinicopathological findings in a six-year-old dog diagnosed with *E.canis*

	Pre Treatment	During treatment (10 days)	End of treatment (30 days)	One month post treatment	Six months post treatment	Ref. interval
Serum protein electrophoresis	**Oligoclonal gammopathy**	-	-	-	-	
C – Reactive protein	**68.8 mg/l**	-	1.6 mg/l	-	-	*0–10 mg/l*
Urine pH	**7**	-	6	6	6	
Urine specific gravity	**1.012**	-	1.036	1.048	> 1.050	
Proteinuria	**3+**	**-**	**3+**	**3+**	**3+**	
UPCR	**4.7**	-	**-**	**5.5**	**1.35**	*< 0.5*
Ehrlichia spp. – antibodies (lateral flow ELISA)	**Positive**	-	**-**	**-**	**-**	
E. canis - antibodies (ELISA)	**110.81 TE**	-	**-**	**-**	**-**	*< 29 TE*
E. canis – antibodies (IFA)	**1:1024**	-	**-**	**-**	**-**	*< 1:32*
E. canis PCR blood	**Positive**	-	Negative	Negative	Negative	
E. canis PCR bone marrow	Negative	-	-	-	-	

Bold text indicates abnormal values

*ELISA* Enzyme-linked immunosorbent assay, *IFA* Indirect immunofluorescence antibody, *PCR* Polymerase chain reaction, *Ref. interval* Reference interval, *spp.* Species, *TE* Titre equivalents, *UPCR* Urine protein-to-creatinine ratio

Coagulation testing (prothrombin time and activated partial thromboplastin time) was performed to investigate a potential bleeding diathesis, given the history of gingival haemorrhage, and results were within reference intervals. Antigen detection testing for *Angiostrongylus vasorum* (IDEXX Angio Detect™) and a modified Baermann examination were both negative, making *angiostrongylus vasorum* infection as a cause for the bleeding unlikely. C-reactive protein concentration was markedly elevated, consistent with clinically significant inflammation (Table 2). Serum cobalamin and folate were measured to investigate for gastrointestinal malabsorption or maldigestion as contributors to the chronic weight and muscle loss, concentrations were within reference limits.

Point-of-care lateral flow ELISA screening (IDEXX SNAP^®^ 4Dx^®^ Plus Test) for *Anaplasma* spp., *Ehrlichia* spp. and *Borrelia burgdorferi* antibodies, as well *Dirofilaria immitis* antigens yielded a positive result for *Ehrlichia* spp., which was confirmed on repeat testing (Table 2). An ELISA specific for *E. canis* antibodies (Laboklin) demonstrated markedly elevated antibody titre levels (110.81 Titre Equivalents [TE], where values > 29 TE are considered positive) (Table 2). An IFA test for *E.canis* antibody detection performed at North Carolina State Veterinary Hospital Diagnostic Laboratory as part of a Vector-Borne Canine Serology Panel demonstrated a high IFA titre (1:1024) suggestive of an active *E. canis* infection (Table 2). TaqMan^®^ real-time PCR (Laboklin) was also positive, confirming an active *E. canis* infection (Table 2).

Imaging was performed as part of the initial investigation for fever of unknown origin and potential bleeding diathesis, prior to all initial results being available to the clinicians. Thoracic radiographs were unremarkable, while abdominal ultrasonography revealed splenomegaly and hepatomegaly. Splenic cytology demonstrated marked plasma cell hyperplasia with mild hemosiderin accumulation, and hepatic cytology showed diffuse vacuolation, cellular degeneration, moderate-to-marked plasma cell hyperplasia, and moderate lymphocytic inflammation. Fine needle aspirates for cytology of the enlarged peripheral lymph nodes were not performed.

As *E. canis*, would be a highly unlikely endemic infection in an Irish dog, additional testing was performed to exclude more common differential diagnoses for thrombocytopenia, anaemia, proteinuria and hyperglobulinaemia. Antinuclear antibody testing by indirect immunofluorescence at IDEXX was negative, with a titre of < 1:25, making systemic lupus erythematosus unlikely. Bone marrow cytology and histology were undertaken to investigate the bicytopaenia and hyperglobulinaemia associated with plasma cell infiltration of the spleen. Findings included erythroid hyperplasia with ineffective erythropoiesis and evidence of rubriphagocytosis, suggestive of an associative precursor-targeted immune-mediated anaemia. Megakaryocytic hypoplasia was also observed, while the granulocytic lineage appeared unremarkable. Moreover, mild plasmacytosis was inconsistent with a multiple myeloma.

Finally, IFA to detect antibodies against *Leishmania infantum*, *Rickettsia rickettsii*, *Bartonella* spp., *Babesia canis*, and *Babesia gibsoni* at North Carolina State Laboratory were negative, providing no serological evidence of co-infection. Bone marrow PCR for *E. canis* was negative (Table 2). Collectively, these findings supported a diagnosis of chronic-phase *E. canis* infection with prognostically relevant cytopenias.

**Fig. 2:**
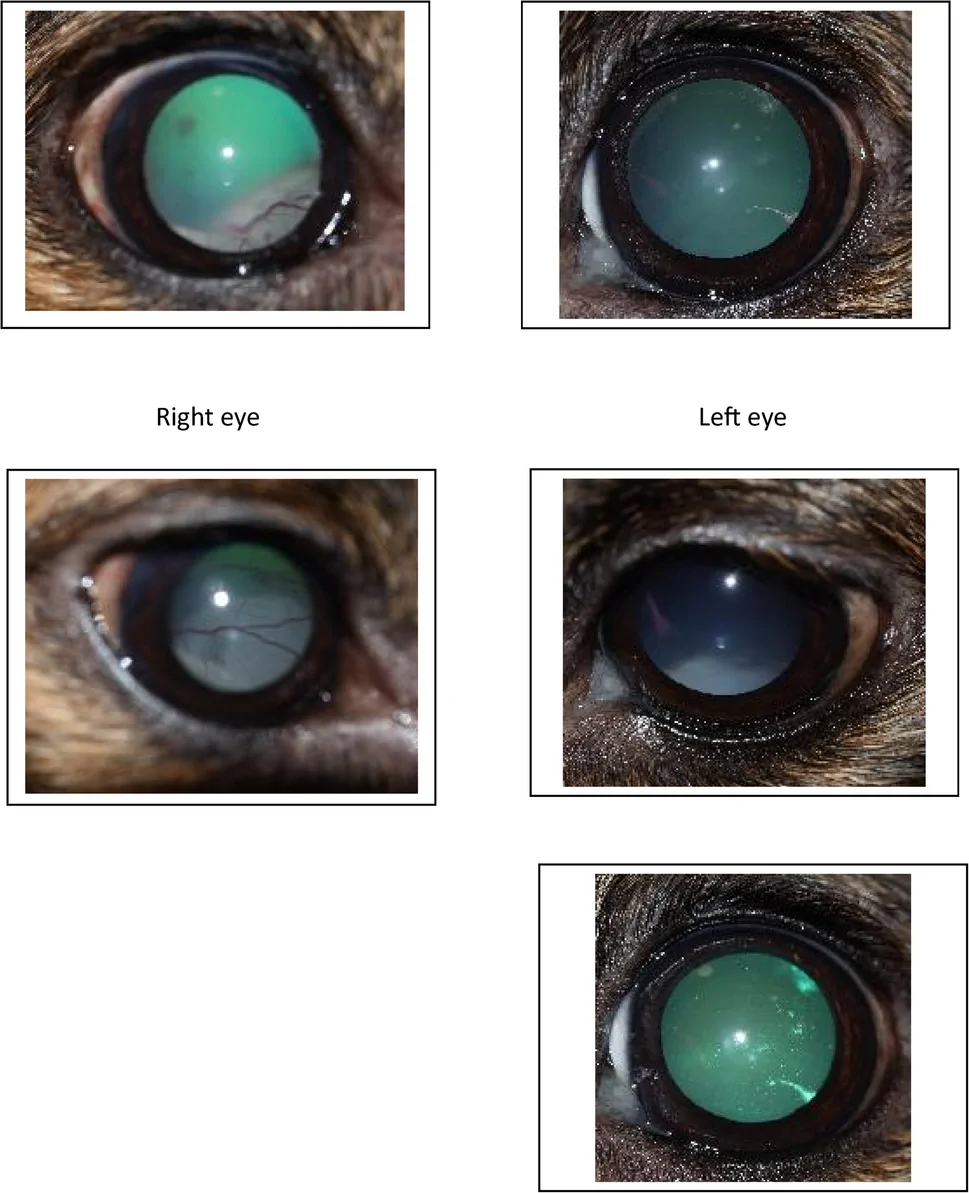
Images showing bilateral partial retinal detachments, chorioretinitis and anterior uveitis in a six-year-old dog diagnosed with E. canis. *Right eye*: partial dorsal and ventral retinal detachment, the ventral retina can be seen pushing against the posterior lens, an incidental region of corneal fibrosis can also be seen. *Left eye*: partial ventral retinal detachment (less severe than right eye), an incidental incipient cortical cataract can be seen

Investigation of the reported ocular abnormalities was performed by a board-certified veterinary ophthalmologist and demonstrated bilateral partial retinal detachments, chorioretinitis and anterior uveitis, ocular abnormalities consistent with, but not specific for, *E. canis* infection (Fig. 2). Examination of the right eye revealed partial dorsal and ventral retinal detachment. There was retinal oedema immediately radiating from the dorsal optic nerve head following the dorsal retinal vasculature. Examination of the left eye revealed a partial ventral retinal detachment. Dorsal retinal oedema was present and was symmetrical with the right eye. Aqueous flare was detected in both eyes. Incidental ophthalmic examination findings included a focal region of corneal fibrosis in the right eye and an incipient cortical cataract in the left eye (Fig. 2).

**Fig. 3:**
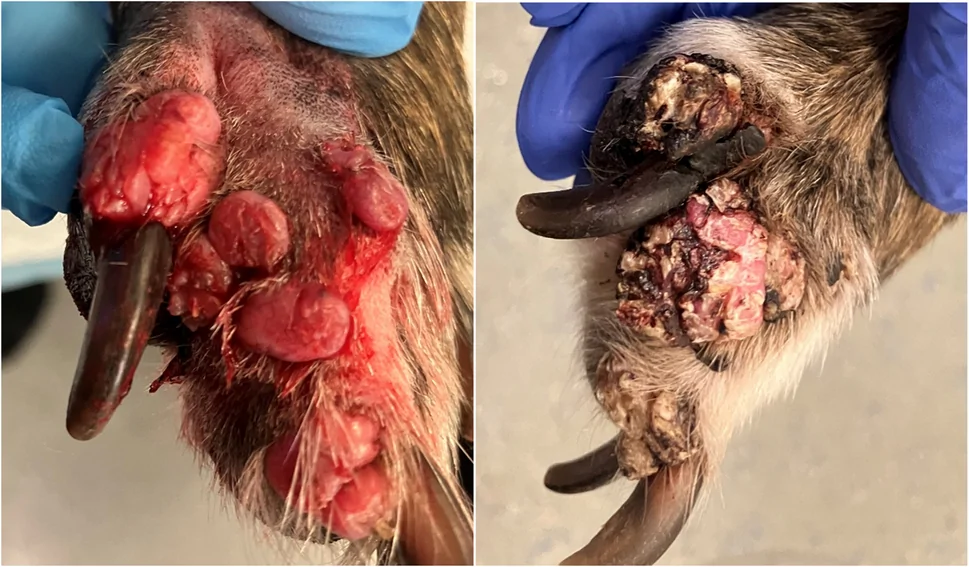
Images showing viral papillomatosis on the paws, which developed during corticosteroid administration in a six-year-old dog treated for E. canis infection

The dog received doxycycline (10 mg/kg orally every 24 h) for four weeks for systemic *E.canis* infection. Clopidogrel (10 mg/kg orally every 24 h) was administered for eight weeks as an antithrombotic given the protein losing nephropathy. A clinical renal diet or antiproteinuric agents were not provided concurrently. For the associated ocular disease, systemic prednisolone was administered at 0.5 mg/kg orally every 12 h and, topical ketorolac trometamol (Acular^®^ 0.5% w/v, Allergan, Ireland) was applied to both eyes every 12 h for ten weeks. During therapy, the dog developed viral papillomatosis on the paws, which was confirmed by histopathology (Fig. 3). Lesions resolved following tapering and discontinuation of corticosteroid therapy.

Treatment resulted in resolution of all associated clinical signs and clinicopathological abnormalities, apart from persistent mild proteinuria (Tables 1 and 2). TaqMan^®^ real-time PCR (Laboklin) for *E.canis* was negative at the end of the course of doxycycline, one month post and six months post treatment (Table 2). Beyond the systemic cure of CME in this dog, a very favourable bilateral visual outcome was achieved. Prompt intervention permitted progressive resolution of chorioretinitis and retinal reattachment, as documented on serial follow-up ophthalmic examinations.

## Discussion and conclusions

This report documents the first confirmed case of suspected endemic *E. canis* infection within Ireland, raising concern for an emerging vector-borne disease in a previously non-endemic region. Historically, serologic testing for *Ehrlichia* spp. in 500 Irish dogs has been uniformly negative; however, one dog with negative serology was subsequently PCR-positive [9]. The positive sample originated from a Bernese mountain dog with no history of travel outside Ireland that was diagnosed with hepatosplenic haemophagocytic histiocytic sarcoma. The absence of seropositivity in that case could be explained by (1) an acute infection without detectable antibodies at the time of sampling, or (2) a false-positive PCR result. Importantly, this dog had received a blood transfusion, raising the possibility that the infection may have been transfusion-acquired. As the dog subsequently died, seroconversion could not be assessed, leaving the validity and origin of the suspected infection uncertain.

At the time our case was diagnosed, we were also aware of a similar presentation in another Irish referral veterinary hospital involving a three-year-old female neutered whippet with acute immune-mediated haemolytic anaemia and thrombocytopenia, in which *Ehrlichia spp.* exposure was suggested by serologic screening. Although the clinical presentation, haematologic abnormalities and favourable response to doxycycline were compatible with *Ehrlichia*-associated disease, diagnostic certainty was limited: PCR testing was negative and quantitative IFA titres were not available, precluding assessment of seroconversion or confirmation of active infection. Accordingly, while this case raised concern for possible autochthonous *Ehrlichia spp.* exposure in another dog with no travel history, the evidence remained insufficient to fully confirm infection. The present case, by contrast, provided confirmatory molecular and serologic evidence of *E. canis* infection in a dog with no travel history, suggesting that transmission may now be occurring within Ireland. This represents a shift from prior assumptions regarding the absence of endemic *E. canis* in the country.

The present case demonstrated comprehensive evidence of ehrlichiosis infection. The clinical, haematologic, biochemical, and serologic findings corresponded closely to the recognised pathophysiology of long-standing *E. canis* infection. The dog’s persistent bicytopaenia (thrombocytopenia and non-regenerative anaemia) was suggestive of chronic disease phase, although chronic ehrlichiosis is more commonly associated with pancytopenia [10, 11]. The underlying cytopenias result from bone marrow suppression, ineffective haematopoiesis, and immune-mediated destruction [10, 11]. The marked hyperglobulinaemia further reflected sustained antigenic stimulation [10]. The primary differential diagnosis for the documented glomerulopathy, characterised by marked glomerular proteinuria, was immune-mediated glomerulonephritis secondary to immune-complex deposition, a complication of advanced disease [10]. However, this diagnosis was not confirmed as renal biopsies were not performed Moreover, the chorioretinitis, retinal detachment, and uveitis were all compatible with previously described ocular manifestations of chronic *E. canis* infection [10, 12, 13].

Serologic and molecular findings supported chronic disease phase interpretation. The high IFA titre (1:1024) was suggestive of chronic infection, in which antibody levels may remain markedly increased, sometimes exceeding 1:600,000 [10]. The positive point-of-care lateral flow ELISA (SNAP^®^ 4DX PLUS) test aligned with its reported sensitivity and specificity (97.8% and 92.3%, respectively) [14]. The positive blood PCR provided definitive evidence of active infection, consistent with reports that *E. canis* DNA becomes detectable 4–10 days after infection [15]. Notably, the sensitivity of blood PCR for chronic ehrlichiosis diagnosis may be reduced, and negative results do not exclude its presence [15, 16]. The negative bone marrow PCR result was unsurprising, given the variable and often low sensitivity of bone marrow sampling, which may detect DNA in as few as 25% of cases, and therefore does not contradict the diagnosis [10, 11].

During treatment, the dog developed viral papillomatosis localized to the paws, confirmed by histopathology. The papillomatosis fully resolved with treatment of the *E. canis* infection and after discontinuation of corticosteroids. Papillomatosis has been reported in dogs with chronic *E. canis* infection and has also been observed in experimentally infected dogs receiving immunosuppressive therapy during the acute or subclinical stages, with lesions regressing following withdrawal of prednisolone and ciclosporin therapy [16, 17].

Doxycycline at 10 mg/kg orally every 24 h for 4 weeks is the treatment for CME [18]. The improvement in clinical signs and haematologic parameters in this case aligned with typical recovery patterns, although full normalisation of chronic-phase abnormalities such as cytopenias and hyperglobulinemia may take months. Moreover, antibody titres may remain high for years and treatment should be guided by clinical response, hematologic improvement, and negative PCR results rather than seronegativity [2, 3, 10]. The decision to use corticosteroids to manage immune-mediated complications, followed by appropriate tapering once papillomatosis developed, reflects current recommendations to limit immunosuppression due to risk of reactivating infection [2, 3, 10]. Predictors of poor prognosis in CME include severe leukopenia (WBC < 0.93 × 10³/µL), anaemia (PCV < 11.5%), prolonged aPTT (> 18.25 s), and hypokalaemia (< 3.65 mmol/L) [19]; notably, these were not prominent features in this case, correlating with the favourable clinical outcome. The complete resolution of concurrent ocular abnormalities in this dog was fitting with a previous study documenting that, with appropriate treatment, a significantly higher proportion of dogs will experience complete resolution of their ocular signs compared with partial or poor responses to treatment [13].

A limitation of this case was the premature discontinuation of clopidogrel after eight weeks of treatment despite persistent marked proteinuria (UPCR 5.5; Table 2), which may have increased the risk of thrombosis [20]. Additionally, neither a renal therapeutic diet nor an antiproteinuric agent, such as an angiotensin receptor blocker, was instituted to manage the persistent glomerular proteinuria. Current guidelines recommend standard antiproteinuric therapy when the UPCR persistently exceeds 0.5 in dogs with glomerular disease, regardless of whether the underlying glomerular injury is primary or secondary [21]. Despite the absence of specific antiproteinuric therapy, treatment of the underlying *E. canis* infection ultimately resulted in a > 50% reduction in UPCR, indicating a substantial improvement in proteinuria.

The detection of *E.canis* infection in a dog with no travel history raises an important epidemiologic question regarding the potential establishment of *R.sanguineus* in Ireland. Although this vector has historically been limited to warmer climates, sporadic reports have documented its presence on a dog in Dublin with no travel history, and two unconfirmed historical cases in Belfast [22, 23]. If transmission in the case discussed occurred via *R. sanguineus*, this may reflect ecological adaptation of the tick, potentially facilitated by climate change, increased pet movement, and importation of infected animals [8]. Moreover, the northern distribution limit of *R. sanguineus* in Europe is largely estimated, as persistent populations may survive at higher latitudes in sheltered environments, such as burrows, kennels, or private gardens, where conditions suitable for survival are maintained [3]. However, no ticks were recovered from the dog, so endemic establishment of the tick cannot yet be confirmed. Experimental studies have also shown that *E. canis* can be transmitted by *Dermacentor spp*. ticks. Although these ticks have not, to our knowledge, been detected in Ireland, they have been reported in northern Europe, including the United Kingdom [9, 24].

Ticks that acquire *E. canis* as larvae retain the organism through the nymphal and adult stages, allowing transmission to new hosts during blood meals [1, 2, 25, 26]. Adult ticks remain capable of transmitting *E. canis* for up to 155 days after acquisition, facilitating long-term persistence of the bacterium during periods of tick inactivity [2, 27, 28]. Age, breed, or sex predilections for *E. canis* infection in dogs have not been established [1–3].

In conclusion, this case underscores the potential for vector-borne pathogens to emerge in regions previously considered free of infection. It highlights the essential role of comprehensive diagnostics and continued epidemiologic surveillance under a One Health framework to detect, prevent, and control evolving tick-borne disease threats in Ireland and beyond.

## Data Availability

No datasets were generated or analysed during the current study.

## Abbreviations

CME: Canine monocytic ehrlichiosis
ELISA: Enzyme-linked immunosorbent assay
IFA: Indirect fluorescent antibody
Kg: Kilogram
µL: Microlitre
µm: Micrometre
Mg/kg: Milligram per kilogram
PCR: Polymerase chain reaction
spp.: Species
TE: Titre Equivalents
W/V: Weight per volume
